# The Relation of Callous–Unemotional Traits and Bullying in Early Adolescence Is Independent from Sex and Age and Moderated by Conduct Problems

**DOI:** 10.3390/brainsci11081059

**Published:** 2021-08-12

**Authors:** Gennaro Catone, Luisa Almerico, Anna Pezzella, Maria Pia Riccio, Carmela Bravaccio, Pia Bernardo, Pietro Muratori, Antonio Pascotto, Simone Pisano, Vincenzo Paolo Senese

**Affiliations:** 1 Department of Educational, Psychological and Communication Sciences, Suor Orsola Benincasa University, 80120 Naples, Italy; gennaro.catone@unisob.na.it; 2 Department of Psychology, University of Campania “Luigi Vanvitelli”, 80120 Naples, Italy; almericoluisa@hotmail.com (L.A.); anna.pezzella333@gmail.com (A.P.); vincenzopaolo.senese@unicampania.it (V.P.S.); 3 Department of Translational Medical Sciences, Federico II University, 80120 Naples, Italy; piariccio@gmail.com (M.P.R.); carmela.bravaccio@unina.it (C.B.); 4 Department of Neuroscience and Rehabilitation, Santobono-Pausilipon Children Hospital, 80120 Naples, Italy; pia.bernardo84@gmail.com; 5 IRCCS Stella Maris, Calambrone, 56128 Pisa, Italy; pietro.muratori@fsm.unipi.it; 6 Department of Mental and Physical Health and Preventive Medicine, University of Campania “Luigi Vanvitelli”, 80120 Naples, Italy; prof.antoniopascotto@gmail.com

**Keywords:** callous–unemotional traits, conduct problems, bullying, cyberbullying, gender

## Abstract

In youths, callous–unemotional (CU) traits and conduct problems (CP) are independently associated with bullying perpetration and these effects are also observed when controlling for sex. Moreover, research indicates that the co-existence of high levels of both CU and CP further increase the risk. Although several studies have examined the relationship between CU traits and traditional bullying, few have also included a measure of cyberbullying and very few of them have focused the early adolescence. The aim of this study was to replicate and extend these findings in a large sample of Italian early adolescents considering both traditional and cyberbullying behaviors. Data were extracted from the Bullying and Youth Mental Health Naples study (BYMHNS) which included 2959 students of 10–15 years of age. CP, CU traits, traditional bullying behaviors, and cyberbullying behaviors were assessed by multi-item self-report scales. As expected, we replicated the significant and specific association between CU traits and traditional bullying, extending the findings to cyberbullying. In addition, in the latter case the effect was moderated by CP. The theoretical and clinical implications of these results were discussed.

## 1. Introduction

### 1.1. CU Traits

Callous–unemotional (CU) traits identify a psychological construct characterized by the absence of concern for the feelings of others, lack of guilt or remorse feelings, lack of empathy, superficial or inadequate affectivity, and lack of concern for the consequence of one’s actions [1,2,3,4,5,6]. In the literature, it has been shown that CU traits constitute the affective dimension of psychopathy in adults [7] and that in children and adolescents the presence of high levels of these traits is associated with a higher risk of deficits in affective processing and future development of antisocial behaviors and other negative outcomes [8,9,10,11,12,13]. Moreover, the latest version of the Diagnostic and Statistical Manual of Mental Disorders (DSM-V) includes CU traits as a specifier for the diagnosis of conduct disorder, designating a group characterized by “limited pro-social emotions” (LPE) [14].

Although CU traits have been mainly studied in populations of children and adolescents with conduct disorders, there is growing evidence that CU traits should be also considered in non-clinical samples given that high scores on this dimension can be observed in individuals not showing evident conduct problems [12,15,16,17,18,19]. For example, Pardini and Byrd [20] highlighted that children (mean age = 10.31; *SD* = 0.72; male = 47.9%) with higher CU traits have a unique and particular deviant social pattern that is not common to all aggressive children. Indeed, compared to children with aggressive behaviors but without high CU traits, those with high levels of CU traits use more aggression to dominate others, have more difficulty in anticipating discomfort and suffering in others, and show less concern for the others or for the consequences of their behavior. Moreover, data showed that independently of general conduct problems, CU traits are positively associated with aggression in both children and adolescents [15,21,22,23,24,25,26], including bullying behaviors [24,26,27,28,29,30,31,32]. Thus, high CU traits could be considered a general risk factor for the development of particularly severe, persistent, and treatment-resistant forms of conduct disorder [33].

### 1.2. Bullying

Among the various aggressive behaviors, bullying is one of the most studied. According to Olweus [34], bullying is defined as an intentional, reiterative, and aggressive behavior that an individual or group may make toward a person and that denotes an asymmetrical relationship, characterized by an imbalance of physical, intellectual, or strength power [34,35,36,37,38]. Prevalence of bullying varies depending on the study selected and worldwide. In this sample, we found a prevalence of traditional bullying victimization and perpetration ranging from 11.4% to 40.7% and 5.1% to 22%, respectively, depending on the assessment method [39] and a prevalence of cyberbullying victimization perpetration of 13.5/5.2% [40]. Recently, an Australian systematic review and meta-analysis detected a 12-month prevalence of traditional bullying victimization of 15.17% and perpetration of 5.275%, and a cyberbullying victimization and perpetration of lifetime prevalence of 7.02% and 3.45% [41].

Bullying is a widespread phenomenon that occurs in different social contexts and, more recently, in the online context. Aggressive bullying behaviors represent a serious risk factor for the psychological well-being of children who are victims [42], and for this reason bullying is considered a serious social problem in many countries [43], including in Italy [44,45,46].

In recent years, many studies have focused on intervention program for bullying behaviors. Zych et al. conducted a systematic review on community, school, family, peer, and individual protective factors that could be enhanced in bullying and cyberbullying preventive programs. They found that self-oriented personal competencies were protective against victimization, whereas good academic performance and other-oriented social competencies were protective against perpetration. Good peer interaction was a protective factor against the behavior of bully/victim and a low use of technology in terms of frequency was protective against cyberbullying [47]. In the same direction, Hinduja and Patchin found the construct of resilience a strong protective factor against bullying and cyberbullying behaviors [48]. Several intervention programs have been recognized as effective in reducing bullying behavior in school and other contexts [49,50,51]. 

Although several factors responsible for bullying behavior perpetration have been highlighted in the literature, it has recently been shown that CU traits seem to have a specific relationship with this behavior that would be independent of the sex, age, and manifestation of general conduct problems. Several international studies have shown that the CU trait is positively correlated with the perpetration of direct bullying [11,32,52] and that antisocial youth with high CU traits were more likely to perpetrate bullying than antisocial youth with low CU traits [15]. Furthermore, evidence has indicated that youths with high CU traits are less likely to respond positively to typical bullying interventions and show less concern for punishment, suggesting that anti-bullying intervention programs should take into account these traits [53].

### 1.3. Previous Studies on the Relationship between CU and Bullying

Even though the link between CU traits and bullying has been confirmed by some studies, the literature on the strength of their association and the role of factors influencing it (e.g., sex, age, conduct problems) is still scarce; moreover, it is very important to further study the relationship between CU traits and bullying in a valid way, by taking into account some important methodological aspects such as the assessment methodology and the context considered. Regarding the former, as stressed by several authors, for the detection of different bullying behaviors it is preferable to adopt a multi-item approach [54]. Indeed, a recent study [39] that compared single-item and multi-item measurements confirmed that the latter methodology offers a more valid detection and better captures the different degrees of bullying. As regards the context, it is worth noticing that most of the studies in the literature have investigated the process influencing bullying in traditional face-to-face contexts (e.g., school), whereas it is important to emphasize that with the wide spread of electronic communication and the use of computers and/or mobile phones by young people, bullying is no longer restricted to the face-to-face interactions but it is also observed in the virtual contexts [55]. Smith and colleagues introduced the term cyberbullying [56] to define bullying carried out through the use of digital technologies and the Internet (e.g., mobile phones, messaging platforms, social media, gaming platforms). Though some studies have provided evidence of an overlap between traditional bullying and cyberbullying [57,58], it has also been shown that cyberbullying differs from traditional bullying because it is characterized by the absence of spatio-temporal boundaries and the possibility of the anonymity of the perpetrator [59]. This latter aspect is particularly relevant given that the anonymity offered by the Internet leads adolescents to express themselves more recklessly and aggressively online than they would in face-to-face interactions [60].

As regards the negative effects of bullying and cyberbullying, if from one side data indicated that both are associated with the same consequences in the victims, such as anxiety, depression, substance abuse, suicidal ideation, and psychosis [61,62,63,64], on the other side, a study directly comparing the impact of traditional face-to-face bullying and cyberbullying on victims reported that the latter is associated with more frequent and intense anxiety and depressive symptoms than the former, particularly in terms of social anxiety [65]. Therefore, it is particularly important to also explore factors that may increase or decrease the risk of cyberbullying perpetration in adolescence.

Studies describing the association between CU traits and cyberbullying in adolescents showed that the two dimensions are significantly and positively associated [29,66,67,68], and that adolescents with high CU scores who manifest cyberbullying behavior tend to ignore the fear and the distress of the victims [69,70], thus increasing the risk in victims of developing symptoms of psychological distress [65,71,72,73,74]. For this reason, it is particularly important to study and prevent cyberbullying as it is easier to carry out than traditional bullying and leads to greater personal and social consequences [65]. This latter consideration is clearer if it is considered in the perspective of the interpersonal acceptance-rejection theory (IPARTheory) [75,76,77]. Indeed, according to the IPARTheory, the quality of individuals’ interpersonal relationships, i.e., perceived acceptance–rejection, influences the general psychological adjustment and the expression of internalizing and externalizing problems. Therefore, because victims of traditional bullying and cyberbullying experience rejection from peers, they could manifest a psychological maladjustment and this, in turn, could lead them to engage inappropriate, aggressive, and problematic behaviors. In other terms, the expression of these negative behaviors increases the risk of its spreading since those who undergo bullying experiences could be led in turn to perpetrate them on others [78], for example using the virtual dimension for revenge for victimization [79,80]. Therefore, it is critically important to understand how much individual characteristics such as CU traits are specifically related to bullying behaviors and to what extent they may represent a general risk factor, to design targeted intervention programs aimed at reducing these phenomena, thus preventing their consequences.

In summary, the analysis of the literature on the relationship between bullying and CU traits indicates a need to replicate these studies by considering very large samples, to attain more replicable estimate of the effect sizes, and by considering bullying in both its traditional (face-to-face) and online (cyberbullying) forms, to better understand the extent to which the relationship is specific and whether it is moderated by other factors such as sex, age, the presence of conduct problems, and the context. Most research, indeed, focused exclusively on a single context of bullying behaviors (see for example [29,52]). Only very few studies have explored the relationship between CU traits and bullying in adolescence considering both the traditional and the cyber forms. Among these, Orue and Calvete [67] showed a significant predictive value of CU traits for both traditional and cyberbullying in a sample of 765 Spanish adolescents aged 14–18 years, and an Italian study, conducted on a sample of 540 subjects aged 10–16 years [27], showed that the presence of CU traits increased bullying behavior in both traditional and cyberbullying contexts.

Hypothesis for the present study: Starting from the abovementioned considerations, the aim of the present study was to replicate and extend the data present in the literature [27,52,67] responding to the need to verify the relationship between the presence of CU traits and bullying behavior on a very large sample, by using multi-item standardized measures and considering different bullying contexts. In particular, we wanted to investigate the predictive and specific role of CU traits independently of the sex, age, and presence of general conduct problems on bullying behaviors. To verify to what extent similar processes regulate both face-to-face and cyberbullying behaviors, both contexts of bullying were considered. In line with the previous literature, we expected to find a specific and significant relationship between CU traits and both forms of bullying and that this effect would be moderated by the conduct problems. In addition, a further objective of the study was to test the moderating effect of the sex and the age factors.

## 2. Methods

### 2.1. Participants

The data considered in this study were extracted from the Bullying and Youth Mental Health Naples study (BYMHNS), a larger cross-sectional study based on a sample of students gathered in the metropolitan city of Naples and in the surrounding areas. The data were collected during the 2015/2016 school years. Twelve schools comprising a total of 4444 students were contacted and agreed to participate. The final total sample of participants consisted of 2959 students of which 1426 (48.2%) were females and 1533 (51.8%) were males; with 44% of the participants belonging to schools in the city of Naples and 56.1% to those in the surrounding areas. As regards the class, 1048 (35.4%) students attended the first grade, 995 (33.6%) the second grade, and 916 (31%) the third grade. The mean age was 11.84 years (*SD* = 0.97, range: 10–15 years).

### 2.2. Procedure

Data were collected through the administration of self-assessment scales to obtain measures of traditional bullying, cyberbullying, and other demographics and psychopathological information. For each school, meetings were held with the headmaster and teachers to provide information about the study. In addition, the parents of the pupils received informed consent in which they express their agreement to the participation of their children in the research. During the administration of the protocol, which happened in the usual classroom and lasted about 1 h, the presence of at least one researcher was guaranteed to provide explanations and to answer any questions from the students. We had very few missing data (<1%) that were handled with means replacing. The Ethics Committee of the University of Campania “Luigi Vanvitelli” approved the study protocol (No. 500 of 29/04/2016). For more information about the whole project please refer to Catone et al. [39].

### 2.3. Measures

#### 2.3.1. Conduct Problems

To attain a measure of general behavioral problems, responses to the conduct problems subscale of the Italian self-report version of the Strength and Difficulties Questionnaire for age 4–17 (SDQ) [81] were considered. The SDQ is a short self-report questionnaire useful for assessing the level of general psychopathology related to the last six months both in clinical and research settings [82]. It consists of 25 items divided into 5 subscales of 5 items each: emotional problems (no reversed); conduct problems (1 reversed); hyperactivity problems (2 reversed); peers problems (2 reversed); pro-social behavior (no reversed). In this study, only responses to the conduct problems subscale (e.g., “I get very angry and often lose my temper”) were considered. Responses were collected on a 3-point Likert type scale: “not true” = 0, “somewhat true” = 1, “certainly true” = 2. A total score was computed for each participant (ranges: 0 to 10), with higher scores indicating higher conduct problems (CP). Cronbach’s Alpha = 0.639; ω_t_ = 0.707.

#### 2.3.2. Callous–Unemotional Traits

To measure callous and unemotional (CU) traits, the Italian 22-item version of the Inventory of Callous–Unemotional Traits (22-item ICU) [27,83] was administered. The 22-item ICU [23] evaluates a general callous–unemotional dimension and three sub-dimensions: callousness (9 items; 1 reversed), which refers to lack of empathy, remorse and guilt (e.g., “I do not care who I hurt to get what I want”); unemotionality (5 items; 3 reversed), indicating absence of emotional activation and expressiveness (e.g., “I do not show my emotions to others”); and uncaringness (8 items; all reversed), which is disinterest in the feelings of others and in the performance of daily activities (e.g., “I work hard on everything I do”). The score for each item is calculated on a 4-point Likert scale and ranges from 0 (“not at all true”) to 3 (“definitely true”). A confirmatory factorial analysis carried out on the total sample confirmed that the best fitting factor structure identifies a general callous–unemotional factor and three specific factors, χ^2^ (206) = 1424.13, *p* < 0.001, *RMSEA* = 0.045, 95% *CI* [0.043; 0.047], *CFI* = 0.794, *SRMR* = 0.046, *N* = 2959. Therefore, a total score was computed for each participant (ranges: 0 to 72), with higher scores indicating higher CU traits. Cronbach’s Alpha = 0.697; ω_t_ = 0.692.

#### 2.3.3. Traditional Bullying

To measure the traditional bullying perpetration, the Italian version of the bully subscale of the Illinois Bully Scale (IBS-B) [39] was administered. The Illinois Bully Scale [84] is a self-reported scale that includes 18 items divided into 3 subscales: bully (9-item; e.g., “I annoyed other students”), victim (4-item; e.g., “Other students beat and pushed me”), and fighting (5-item; e.g., “If someone beats me firstly, I will beat him/her”). In this study, for each item of the IBS-B, participants were asked to indicate the frequencies with which they carried out the described behavior. Responses were collected on a 5-point scale, which considered the following alternatives: “never” = 0, “1 or 2 times” = 1, “3 or 4 times” = 2, “5 or 6 times” = 3, “7 or more times” = 4. The good psychometric properties of the Italian IBS-B have been described in Catone et al. [39]. As indicated by Espelage and Holt [84] a total score of traditional bullying perpetration was computed for each participant (ranges: 0 to 36), with higher scores indicating higher self-reported bullying behaviors. Cronbach’s Alpha = 0.824; ω_t_ = 0.859.

#### 2.3.4. Cyberbullying

To measure the cyberbullying perpetration [40], the Italian version of the Smith’s cyberbullying scale (SCBS) was administered. The SCBS [56] is a self-report scale assessing cyberbullying behaviors by considering seven different media (7-item): text messaging; pictures/photos or video clips; phone calls; email; chat rooms; instant messaging; and websites. Participants were preliminarily presented a definition of cyberbullying behaviors then were asked to indicate for each media if they bullied others through it during the last year. Responses were collected on a 5-points scale that considered the following alternatives: “never” = 0; “only once or twice” = 1; “two or three times a month” = 2; “about once a week” = 3; “several times a week” = 4). Although the Italian adaptation of the scale has been already used, its psychometric characteristics have not been described. For this reason, the dimensionality and reliability of the SCBS were preliminarily verified before running the main analyses of this study. A confirmatory factorial analysis carried out on the total sample confirmed the unidimensional structure of the scale, χ^2^(14) = 205.31, *p* < 0.001, *RMSEA* = 0.068, 90% *CI* [0.060; 0.076], *CFI* = 0.976, *SRMR* = 0.026, *N* = 2959; whereas the reliability analysis showed an adequate value, Cronbach’s Alpha = 0.795; ω_t_ = 0.889. Therefore, as indicated by Smith and colleagues [56], a total score of cyberbullying perpetration was computed for each participant (ranges: 0 to 28), with higher scores indicating higher self-reported cyberbullying behaviors.

### 2.4. Statistical Analyses

Prior to carrying out the main analysis, the descriptive statistics were computed to describe considered variables: demographics (sex, age, class, school), conduct problems (CP), CU traits, traditional bullying (IBS-B), and cyberbullying (SBCS) behaviors. Descriptive statistics are reported into Table 1. Data indicated that traditional bullying (IBS-B) and cyberbullying (SBCS) perpetration scores presented a severe deviation from non-normal distribution as indicated by skewness and kurtosis, and were therefore normalized by adding a constant of 1 and applying a logarithmic transformation [85]. All the analyses were performed on the transformed variables, but for descriptive purposes, untransformed data were used to report descriptive statistics. Then, correlation coefficients between the variables age, sex, conduct problems, CU traits, traditional bullying, and cyberbullying measures were computed to investigate the bivariate associations. According to Cohen (1988), for Pearson’s r we considered indicative of small, medium, and large effects the values 0.10, 0.30, and 0.50, respectively. Finally, to investigate if the association between CU traits and bullying and cyberbullying perpetration is observed also when controlling for sex, age, and conduct problems, and if it was moderated by the context of bullying or the control variables, two hierarchical multiple regressions were carried out. In each regression, bullying (traditional or cyberbullying) was regressed on the control variables (sex, age, and conduct problems) and the ICU. In both regression models, all variables were included as z-scores, but sex was dummy-coded (males = 1; females = 0). In the first step, the sex and the age were included. In the second step, the conduct problem variable was added. In the third step, the ICU score was added, whereas, in the fourth and last step, the two-way interaction effects were added. When significant, the interaction effects were investigated by applying simple slope analysis and the Johnson and Neyman’s (JN) approach [86] to define the lower and upper values of the moderator for which the effect of the predictor on the dependent variable was significant. All the analyses were performed with R 4.0.4 software and an Alpha level of 0.05 was used for all statistical tests.

## 3. Results

The main descriptive statistics are reported into Table 1.

The results of the correlation analysis showed a strong association between the two measures of bullying (*r* = 0.615, *p* < 0.001) and that both measures were significantly associated with all the considered variables (see Table 2).

In particular, the analysis of the effect sizes showed that the two bullying measures were weakly associated with the control variables sex and age (*r*s < 0.176, *p*s < 0.001), while the association with conduct problems and CU traits was medium (0.292 < *r* < 0.485, *p*s < 0.001). Finally, data showed a medium association between conduct problems and CU traits (*r* = 0.360, *p* < 0.001). Therefore, male adolescents than females, older adolescents than younger, adolescents with higher conduct problems in the last six months and adolescents with higher scores on the CU trait had higher scores in both bullying and cyberbullying perpetration.

Results of hierarchical regressions are reported in Table 3. Results showed a similar pattern of effects for the two considered dependent variables.

### 3.1. Traditional Bullying

The results of the hierarchical regression analysis on the traditional bullying scores confirmed the positive association between the considered variables and the bullying behaviors, indicating that, over and above sex, age, and conduct problems, the CU trait had specific and additive effects on perpetration (*β* = 0.167, *p* < 0.001), although the effect size was small (*R*^2^_diff_ = 0.024, *p* < 0.001) (see Step 3). Moreover, results of the last step (see Step 4) indicated that the effect of CU traits was not moderated by sex, age, or conduct problems.

### 3.2. Cyberbullying

**Figure 1:**
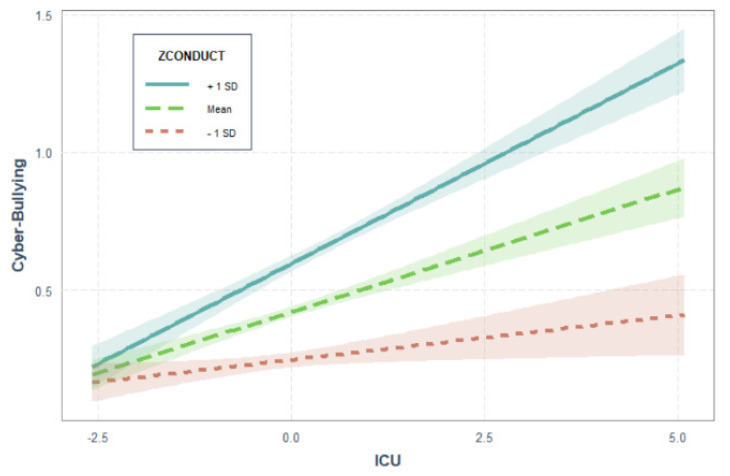
Interaction between CU traits and conduct problems on cyberbullying perpetration.

The results of the hierarchical regression analysis carried out on the cyberbullying scores showed similar results to those observed for the traditional bullying scores. In particular, data confirmed the positive association between the considered variables and the bullying behaviors, indicating that, over and above sex, age, and conduct problems, the CU trait had a specific and additive effects on perpetration (*β* = 0.174, *p* < 0.001). Also in this domain, the observed effect size indicated a small effect (*R*^2^_diff_ = 0.026, *p* < 0.001) (see Step 3). In contrast to previous findings, however, the data showed that the moderate model significantly increased the prediction of differences in the perpetration of cyberbullying (*R*^2^_diff_ = 0.016, *p* < 0.001) (see Step 4). In particular, data showed a significant interaction between conduct problems and CU traits, indicating that the relationship between CU traits and cyberbullying became progressively stronger as conduct problems increased (see Figure 1). Therefore, the co-occurrence of conduct problems and CU traits increased the risk of issuing cyberbullying behaviors. The JN analysis indicated that the effect of CU traits was positive and significant when the conduct problems were higher than −1.08 *SD* from the mean, whereas it was negative and significant when the conduct problems were lower than −2.34 *SD* from the mean. As regards this latter case, it is worth noticing that in this study the range of observed standardized values of conduct problems was from −1.37 to 4.61.

## 4. Discussion

In this study we sought to replicate and extend previous findings on the associations between CU traits and bullying perpetration in a large sample of adolescents, using standardized multi-item measures and by considering both traditional and cyberbullying. Results showed that the two bullying dimensions are remarkably similar and are influenced in a similar way by the variables considered, as indicated by the strong correlation between the traditional and cyberbullying perpetration and the presence of broadly alike correlations between both forms of bullying and conduct problems or CU traits. These results are in line with studies that reported a strong overlap between bullying and cyberbullying [87,88]; e.g., Modecki et al. affirmed that probably the two manifestations are different ways of implementing the same aggressive behavior [89]; whereas Przybylski and Bowes stated that probably cyberbullying almost always occurs together with traditional bullying [90]. However, it is important to emphasize that the data from this study also showed some differences between these two forms of bullying, in line with those authors who have found that the cyber context somehow facilitates such behaviors [59,60,65].

Related to the central point of this study, our results indicated that male, older adolescents, and adolescent with high scores on conduct problems or CU traits had higher scores on measures of traditional and cyberbullying perpetration. Furthermore, the results of the regression analysis indicated that CU traits were specifically associated with bullying perpetration in both traditional and cyber contexts. CU traits significantly increased the traditional bullying perpetration behaviors, and this association was independent of sex, age, or CP. At the same time, data indicated that, over and above sex, age, and conduct problems, CU traits also increased cyberbullying behavior perpetration and that in this latter case the association between conduct problems and cyberbullying perpetration was moderated by CU traits. In other terms, the simultaneous presence of CU traits and conduct problems can be considered a stronger risk factor for the involvement in cyberbullying perpetration.

These results confirm findings from other studies on the positive and significant association between CU traits and bullying aggressive behaviors [11,15,24,26,27,28,29,30,31,32,52]. In particular, Crapanzano et al. [28], in a sample of 284 students (age range 9–14 years), found a correlation between roles of perpetrators and conduct problems, CU traits, positive expectancies for aggression, and low levels of pro-social behavior. Fanti and Kimonis [30], considering a large sample (*N* = 1214), showed that adolescents with high CP and CU traits had a more severe pattern of bullying behaviors than adolescents with lower scores, arguing that the compresence of high CP and CU caused adolescents to pay less attention to the victim’s distress and fear and this, in turn, reduced the possibility of spontaneously inhibiting the behavior. Moreover, these youths were also more likely to foresee that their aggression would result in more positive advantages for them. Golmaryami et al. [31] indicated that both perpetration and victimization were associated with CP, but when CU traits entered in the analysis, the association remained significant only for the group with low levels of victimization. Interestingly, Thornton et al. [26], by considering a sample of 284 ethnically diverse students (age range 9–14 years), showed that CU traits and CP interact in determining bullying proactive aggression; that students with high CP but low CU traits were more likely to express bullying reactive aggression and anger dysregulation, but that low CU traits were found in students who defended bullying victims.

At the same time, our findings slightly differed from those of Viding et al. [52]: While they found, in addition to the main effects that we also observed, that CU and CP interacted in predicting direct and indirect forms of bullying, we found the same pattern but only for cyberbullying. This may be explained in light of the fact that physical or verbal bullying, due to its characteristics of direct confrontation with the victim, was carried out more easily if the perpetrator had a lack of empathy and sensitivity. Cyberbullying can be assimilated more to indirect forms. In these behaviors, there is no direct confrontation with the victim’s fear and distress. This has been called “lack of the emotional reactivity” and several authors have suggested that in cyberbullying, the reward resulting from one’s perpetrated action is not immediate but delayed, and this implies that in an electronic context the perpetration has an intrapersonal purpose (essentially performing the action), rather than an interpersonal one (observing reactions, obtaining a positive outcome [62]). Munoz et al. [32] confirmed these results, showing that those with high CU traits and with low affective empathy were more involved in direct forms of bullying.

These results have some theoretical and practical implications. First, it is particularly important to better understand the factors that underlie aggressive behaviors such as bullying and to distinguish between traditional and digital forms. This can allow us to build psychological and social models capable of having a greater impact on prevention and intervention programs, which in turn can help to prevent some of the negative consequences that in the perspective of IPARTheory [75,76,77] are associated with the experiences of interpersonal rejection: hostility, aggression, passive aggression, or psychological problems with the management of hostility and aggression; emotional unresponsiveness; impaired self-esteem; impaired self-adequacy; emotional instability; and negative world-view. Namely, those personality dispositions which could become stable and that may increase the risk of showing internalizing or externalizing disturbances, respectively facilitating further risk of victimization or the tendency to bullying others as revenge [78,79,80]. Second, children and adolescents with CP and CU traits might request different forms of bullying intervention and prevention programs such as they tend to respond worse to standard treatments [91]. As already suggested by other authors [52], rather than only “educative” or “punitive” programs, a mixed methodology that includes rewards for adequate behaviors, adult or peer mentoring and education with empathy, and social training programs may be more suitable for youth high on CU and CP.

In addition to its merits, several limitations of this study must be also considered. First, our data were correlational and self-reported and this could threaten the internal validity; future studies need to consider different methodologies (e.g., longitudinal) and approaches (e.g., multi-informant) to improve the validity of the data. Our measure of traditional bullying did not differentiate between direct and indirect forms, and this could threaten the construct validity; future studies need to also consider these facets. Third, we did not gather data on socioeconomic status of participants, and thus we could not include this variable in the analysis; future studies need to consider this dimension.

## 5. Conclusions

In conclusion, in this study, we replicated in a large sample of Italian adolescent previous findings indicating that CU traits are significantly and positively associated with bullying behaviors, over and above age, sex, and general conduct problems. Our data confirmed that the specific association between CU traits and bullying behaviors is observed for both traditional and cyberbullying contexts and that in the cyber contexts, in particular, the compresence of CU traits and general conduct problems represents a further risk factor of bullying. Consequently, these results further draw attention to the need to assess the presence of CU traits in order to prevent the bullying phenomenon and, if needed, to design valid and efficacy targeted intervention programs.

## Figures and Tables

**Table 1:** Summary of means, standard deviations, minimum, maximum, skewness, and kurtosis of the considered variables.

Variable	M	SD	Min	Max	Skewness	Kurtosis
Sex	-	-	0	1	-	-
Age	11.84	0.97	10	15	0.04	−0.66
Conduct	2.29	1.67	0	10	0.99	1.14
ICU	21.83	8.08	1	63	0.32	0.07
Traditional-B	3.05	4.14	0	36	3.09	13.91
Cyber-B	0.95	2.17	0	28	6.87	65.40

Note: *n* = 2959. Sex = participants’ sex dummy coding (males = 1; females = 0); Age = age of participants in years; Conduct = conduct problems subscale of the SDQ; ICU = total score of the ICU; Traditional-B = total score of the bully subscale of the Illinois Bully Scale; Cyber-B = total score of the Smith’s cyberbullying scale.

**Table 2:** Summary of intercorrelations (and 95% confidence intervals) for the considered variables.

Variable	1	2	3	4	5
1. Sex					
2. Age	0.018 [−0.02; 0.05]				
3. Conduct	−0.008 [−0.04; 0.03]	0.073 * [0.04; 0.11]			
4. ICU	0.127 * [0.09; 0.16]	0.128 * [0.09; 0.16]	0.360 * [0.33; 0.39]		
5. Traditional-B	0.176 * [0.14; 0.21]	0.101 * [0.07; 0.14]	0.485 * [0.46; 0.51]	0.338 * [0.31; 0.37]	
6. Cyber-B	0.079 * [0.04; 0.12]	0.086 * [0.05; 0.12]	0.376 * [0.35; 0.41]	0.292 * [0.26; 0.32]	0.615 * [0.59; 0.64]

Note: *n* = 2959; Sex = participants’ sex dummy coding (males = 1; females = 0); Age = age of participants in years (z-score); Conduct = conduct problems subscale of the SDQ; ICU = total score of the ICU; Traditional-B = total score of the bully subscale of the Illinois Bully Scale; Cyber-B = total score of the Smith’s cyberbullying scale; * *p* < 0.001. Note that for the correlation between sex and quantitative variables, a point biserial correlation coefficient was computed.

**Table 3:** Hierarchical multiple regression analyses predicting traditional and cyberbullying from sex, age, conduct problems, ICU, and their interaction.

			Bullying			
		Traditional			Cyber	
Predictor	*R* ^2^_diff_	*b* [95% CI]	*β*	*R* ^2^_diff_	*b* [95% CI]	*β*
Step 1	0.045 **			0.021 **		
Sex		0.31 [0.25; 0.37]	0.188 **		0.10 [0.06; 0.14]	0.089 **
Age		0.08 [0.05; 0.11]	0.098 **		0.06 [0.04; 0.08]	0.112 **
Step 2	0.227 **			0.148 **		
Sex		0.32 [0.27; 0.37]	0.192 **		0.11 [0.07; 0.14]	0.093 **
Age		0.05 [0.03; 0.08]	0.063 **		0.05 [0.03; 0.07]	0.084 **
Conduct		0.39 [0.37; 0.42]	0.477 **		0.22 [0.20; 0.24]	0.386 **
Step 3	0.024 **			0.026 **		
Sex		0.28 [0.23; 0.33]	0.171 **		0.08 [0.04; 0.12]	0.071 **
Age		0.04 [0.01; 0.06]	0.046 *		0.04 [0.02; 0.06]	0.066 **
Conduct		0.34 [0.32; 0.37]	0.418 **		0.19 [0.17; 0.21]	0.324 **
ICU		0.14 [0.11; 0.17]	0.167 **		0.10 [0.08; 0.12]	0.174 **
Step 4	0.001			0.016 **		
Sex		0.28 [0.23; 0.33]	0.171 **		0.09 [0.05; 0.13]	0.077 **
Age		0.04 [0.01; 0.06]	0.047 *		0.04 [0.02; 0.06]	0.065 **
Conduct		0.34 [0.32; 0.37]	0.416 **		0.17 [0.15; 0.19]	0.304 **
ICU		0.12 [0.08; 0.16]	0.143 *		0.07 [0.04; 0.10]	0.125 **
Sex × ICU		0.03 [−0.02; 0.08]	0.029		0.03 [−0.01; 0.07]	0.042
Age × ICU		−0.01 [−0.03; 0.02]	−0.010		0.01 [−0.01; 0.03]	0.016
Conduct × ICU		0.01 [−0.01; 0.04]	0.021		0.06 [0.04; 0.07]	0.124 **
Total R2	0.297 **			0.211 **		

Note: *n* = 2959; Age = age of participants in years (z-score); Sex = participants’ sex dummy coding (males = 1; females = 0); Conduct = conduct problem subscale of the SDQ; ICU = total score of the ICU; Traditional = total score of the bully subscale of the Illinois Bully Scale; Cyber = total score of the Smith’s cyberbullying scale * *p* < 0.01; ** *p* < 0.001.

## Data Availability

The dataset that support the findings of this study is available from the corresponding author upon reasonable request.
